# Outcomes After Being Lost to Follow-up Differ for Pregnant and Postpartum Women When Compared With the General HIV Treatment Population in Rural South Africa

**DOI:** 10.1097/QAI.0000000000002413

**Published:** 2020-06-10

**Authors:** David Etoori, Francesc Xavier Gomez-Olive, Georges Reniers, Brian Rice, Jenny Renju, Chodziwadziwa W. Kabudula, Alison Wringe

**Affiliations:** a Department of Population Health, London School of Hygiene and Tropical Medicine, London, United Kingdom;; b MRC/WITS Rural Public Health and Health Transitions Research Unit (Agincourt), School of Public Health, Faculty of Health Sciences, University of Witwatersrand, Johannesburg, South Africa;; c Department of Public Health Environments and Society, MeSH Consortium, Faculty of Public Health and Policy, London School of Hygiene and Tropical Medicine, London, United Kingdom; and; d Department of Epidemiology and Biostatistics, Kilimanjaro Christian Medical University College, Moshi, Tanzania.

**Keywords:** HIV, lost to follow-up, mother-to-child transmission, South Africa, patient outcome assessment, retention

## Abstract

Undetermined attrition prohibits full understanding of the coverage and effectiveness of HIV programs. Outcomes following loss to follow-up (LTFU) among antiretroviral therapy (ART) patients may differ according to their reasons for ART initiation.

**Setting:**

We compare the true outcomes of adult patients previously identified as LTFU by reason for ART initiation in 8 health facilities in north eastern South Africa.

**Methods:**

Adult HIV patient records were linked to health and demographic surveillance system (HDSS) data from 2014 to 2017. Outcomes of adults categorized as LTFU (>90 days late for the last scheduled clinic visit) were determined through clinic and routine tracing record reviews, consultation of HDSS data, and supplementary tracing. We calculated the proportion of patients per outcome category and performed competing risk survival analysis to estimate the cumulative incidence of death, transfer, migration, ART interruption, and re-engagement following LTFU.

**Results:**

Of 895/1017 patients LTFU with an outcome ascertained, 120 (13.4%) had died, 225 (25.1%) re-engaged, 50 (5.6%) migrated out of the HDSS, 75 (8.4%) were alive and not on treatment, and 315 (35.1%) transferred their treatment. These outcomes varied by sex and pregnancy status at ART initiation. Mortality was less likely among pregnant women, patients with higher baseline CD4, and more likely among older patients.

**Conclusions:**

Patient survival and transfers to other facilities are considerably higher than those suggested in earlier studies. Outcomes differ for women who were pregnant or postpartum when initiating ART, with this population less likely to have died and more likely to have migrated.

## Abstract 2

Supplemental Digital Content is Available in the Text.

## INTRODUCTION

As HIV programs in sub-Saharan Africa have expanded, emphasis has been put on initiating patients on antiretroviral therapy (ART) as early as possible in the course of HIV infection.^1,2^ Eligibility for ART has changed since the adoption of Option B+ which made all pregnant and postpartum women eligible for ART as soon as they tested HIV positive and “Treat all” which extended this eligibility to all people living with HIV.^3^ Although ART initiation rates among people diagnosed with HIV have increased,^4–6^ many programs have experienced high attrition rates, especially among women who initiate ART for prevention of mother-to-child transmission of HIV (PMTCT).^7^ Many of these patients are classified as lost to follow-up (LTFU), a general term for unknown outcomes of patients who have not returned for a scheduled clinic visit. LTFU is often an amalgamation of “silent” (undocumented) clinic transfers, treatment interruptions or stoppages, and deaths,^8–12^ which are challenging to accurately document using routine reporting mechanism.^13–15^

Misclassification of patients as being LTFU can lead to as much as a five-fold underestimation of retention and deaths.^16^ Understanding true outcomes among patients who are reported as LTFU is important to accurately monitor and report on indicators for national ART programs and better target tracing efforts.^10^ Accurate mortality estimates are also important for parameterizing epidemic projections in software programs such as the UNAIDS Spectrum package.^17^

A systematic review of HIV patient tracing studies conducted in sub-Saharan Africa from 2001 to 2012 reported that 39% of patients documented as LTFU in clinic records had died, 18.6% had self-transferred to other HIV clinics, and 28.6% had stopped ART but were still alive.^12^ An earlier review covering studies in sub-Saharan Africa undertaken between 2004 and 2008 reported that 42% of patients documented as LTFU in HIV clinics had died.^18^

These 2 reviews were conducted in the earlier stages of sub-Saharan African ART programs when ART patient profiles included a higher proportion with severe immunosuppression at treatment initiation and before universal ART for HIV-positive pregnant women (Option B+) had been introduced.^19^ In addition, decentralization of ART programs means ART can be provided closer to patients' homes,^12^ which may have increased the number of “silent” transfers taking place within these programs. Furthermore, the proportion of pregnant and postpartum women in ART programs has increased since the adoption of Option B+. This population differs from the general adult population on ART in several ways that are likely to affect the true outcomes among those LTFU, yet few studies have traced women LTFU from PMTCT programs.^20^ First, ART initiation eligibility criteria for pregnant women have included higher CD4 counts in many settings over the past decade, such that on average they are more likely to initiate treatment while still asymptomatic.^21^ In addition, childbirth is a risk factor for default from treatment programs^22,23^ for reasons including postpartum depression or out-referral from PMTCT programs after delivery.^24–26^

With recent randomized control trials of universal test and treat showing modest and mixed results regarding reducing HIV incidence,^27–29^ it is imperative that we understand outcomes among nonadherent patients including those LTFU. This will help to develop and direct innovative ways to identify and reach those who have truly disengaged from care. In this context, we conducted a tracing study in Agincourt in rural north-eastern South Africa to ascertain the true outcomes of patients who were LTFU, disaggregated by whether they were pregnant or postpartum when initiating ART (PMTCT) or not, to better understand the outcomes of this group and compare them to the adult ART population who met other criteria for ART initiation.

## METHODS

### Setting

The Agincourt Health and Demographic Surveillance System (HDSS) is located in Mpumalanga province in rural north-eastern South Africa. Established in 1992, the site is approximately 475 square kilometers and has conducted annual demographic surveys within the HDSS population to capture births, deaths, and migrations since 1999.^30,31^ In 2013, HIV prevalence in the HDSS population aged 15 years or older was estimated at 19.4%^32^

The HDSS also collects verbal autopsy (VA) data to ascertain probable causes of death.^33^ In brief, a structured interview was conducted with people who were closely related to or cared for the deceased during the final illness and could report on symptoms and signs they observed during this period. The interview was conducted using a locally validated tool, in the local language. Until 2010, 2 medical doctors independently reviewed the data to assign a cause of death based on international classification of diseases (ICD-10) conventions,^34^ with a third doctor used in the event of a lack of consensus. The cause was coded “undetermined” if this failed to yield any agreement.^30,35^ Since 2011, causes of death are assigned using the InterVA-4 probabilistic model.^36^

There are 5 primary health facilities and 3 secondary community health centers located within the Agincourt HDSS, all of which offer HIV services including testing and treatment. All health facilities routinely trace patients that are late for a scheduled appointment, with some clinics receiving tracing support from 2 nonprofit organizations, Right-to-Care (RtC) (6 facilities) and Home-Based Carers (HBC) (7 facilities). Routine tracing is described in detail elsewhere.^37^ Briefly, tracing procedures are triggered once a patient is more than 5 working days late for a scheduled visit and usually involves 2 steps, 3 phone calls, and a home visit if the phone calls do not yield a satisfactory outcome. Patients are considered LTFU if they have not returned to the clinic 90 days after their scheduled visit.

In 2014, an initiative was started to identify registered HDSS residents when they visited local health facilities. The point-of-contact interactive record linkage (PIRL) matches chronic care (HIV, diabetic and hypertensive) patient information at the health facility to their HDSS record. This is done in the presence of the patient to resolve any indecision about their identity in the event of multiple resident matches.^38^

### Record Review and Tracing Study

Using the PIRL database, we identified patients who were more than 90 days late for a scheduled HIV clinic appointment on August 15, 2017 at any of the 8 health facilities located in the Agincourt HDSS. Patients were included in the cohort if they were 18 years or older, had ever declared residency in the HDSS, and had enrolled in HIV treatment after PIRL was established at the health facilities.

Patients who had not yet initiated ART were excluded from our analyses because they did not have a next scheduled visit and as such it was impossible to determine whether they were LTFU or just visited the clinics less frequently. Furthermore, this population would not be comparable to patients who had potentially accrued some benefits from taking ART.

Patients were followed up to ascertain whether they were still alive and still on treatment. Trained fieldworkers conducted a thorough record review, on a case-by-case basis, to resolve each patient outcome by comparing the list of patients LTFU against (1) TIER.Net (the electronic medical records database for health facilities in South Africa)^39^ (2) paper-based patient clinic files, and (3) logbooks kept by RtC and HBC. The PIRL database was also reviewed for duplicate patients who were then considered silent transfers. Residency and vital status were also checked in the HDSS demographic surveillance database.

Home-Based Carers conducted a further home visit for all patients without an outcome resolution (ie, no definitive outcome after the record review and for whom routine tracing had not previously been done). For all patients remaining LTFU, searches were undertaken in TIER.Net databases of clinics in close proximity to their residence to capture any further silent transfers (see Supplementary Figure 1, Supplemental Digital Content, http://links.lww.com/QAI/B486).

### Definitions

A patient was considered to have died if they were reported as deceased in their patient file or in TIER.Net or if they were reported to have died through HDSS surveillance data.

A patient was considered to have re-engaged in care if they were found to still be in care at the same clinic where they initiated treatment but were >90 days late for their last appointment.

A patient was defined as having transferred if they had either reported taking treatment at another clinic, if the clinic at which they initiated ART had communicated with and ascertained their transfer to another clinic, or if there was a record of them collecting treatment from another clinic within the Agincourt HDSS.

Patients were defined as having migrated if they were recorded as such (movement outside the study area) through the HDSS, the migration event happened after their last clinic visit and there was no evidence of them taking treatment at another clinic.

A patient had stopped ART if they had been found and reported that they stopped ART, denied their HIV status or refused to return to the clinic.

A patient was alive with ART status unknown if additional tracing yielded no definitive outcome, but they were found to still be alive through the most recent demographic surveillance round, with a surveillance date after their last clinic visit.

A data error was a situation where a patient was <90 days late for their next scheduled appointment but was erroneously classified as LTFU.

### Statistical Analyses

Counts and proportions were calculated for socio-demographic, baseline clinical characteristics, patient tracing outcomes, and VA causes of death. A Pearson's χ^2^ test was used to compare categorical variables.

Competing risk survival analysis methods were used to estimate the cumulative incidence of death, transfer, migration, ART stoppage, and re-engagement following loss to follow-up (LTFU). Follow-up time began on the date of each patient's last recorded clinic visit as we suspected that some outcomes especially deaths would occur closely following a last visit and before patients would have been categorized as LTFU. Using these cumulative probabilities, status plots were produced stratified by sex, pregnancy status at ART initiation, and baseline CD4.

A Cox regression model was used to determine the factors associated with death, with all other outcomes considered to be right-censored. Bivariate analyses were conducted with a priori selected variables that had been shown to be associated with death in previous studies.^18,40–42^ All variables with *P* < 0.1 were included in the multivariable Cox regression model. A parsimonious model was achieved using Wald tests. All analyses were conducted using Stata 15.^43^ All models accounted for clustering at the clinic level and used robust standard errors.

### Ethics

Ethical approval was obtained from the London School of Hygiene and Tropical Medicine, the University of Witwatersrand, and the Mpumalanga Department of Health.

## RESULTS

### Population Characteristics

Over the study period, 4089 patients were added to the PIRL database and met the inclusion criteria. Of these 4089, 1325 (32.4%) met the LTFU criteria and were eligible for inclusion in the record review and tracing study. Of these 1325 patients, 166 (12.5%) did not have an ART initiation date. Further investigation of these 166 patients found 46 (27.7%) had initiated ART after record linkage, 59 (35.5%) had not yet initiated ART, and 61 (36.7%) had initiated ART before record linkage began. These 61 patients and the 59 non-ART patients were excluded from further analyses. Of the remaining 1205 patients, 188 (15.6%) were misclassified as LTFU due to data errors (missed clinic visits in the PIRL database) and were excluded from further analysis (see Supplementary Figure 2, Supplemental Digital Content, http://links.lww.com/QAI/B486). Analyses of these 188 patients to evaluate the utility of routine tracing are presented in supplementary information (see Supplementary information 1, Supplemental Digital Content, http://links.lww.com/QAI/B486). The remaining 1017 patients were 91–1188 days late (see Supplementary Figure 3, Supplemental Digital Content, http://links.lww.com/QAI/B486).

Of the 1017 remaining patients, 280 (27.5%) initiated ART for PMTCT, 767 (75.4%) were women and 849 (83.5%) linked to an HDSS record (Table 1). Pregnant women were younger with a median age of 29 years (IQR: 25, 33) compared with nonpregnant women, 33 years (IQR: 28, 42) and men, 41 years (IQR: 34, 48). Of 280 patients who initiated ART for PMTCT, 52 (18.6%) had a baseline CD4 <200 cells/µL compared with 193 of 487 (39.6%) nonpregnant women and 146 of 250 (58.4%) men. None of the patients who initiated ART for PMTCT with baseline CD4 <200 cells/µL were categorized as WHO stage III/IV compared with 53 of 193 (27.5%) nonpregnant women and 45 of 146 (30.8%) men. Furthermore, 5.0% of women who initiated treatment for PMTCT had a CD4 less than 100 cells/µL compared with 21.8% of nonpregnant women and 34.4% of men. The main reason for ART initiation for nonpregnant patients was CD4 count criteria (74.5%) (Table 1).

**TABLE 1.** Patient Demographic and Clinical Characteristics, and Final Outcomes Disaggregated by Pregnancy Status at ART Initiation

	LTFU	Pregnant Women	Nonpregnant Women	Men
	1017	280	487	250
	N (%)	N (%)	N (%)	N (%)
Age				
18–29	333 (32.7)	150 (53.6)	157 (32.2)	26 (10.4)
30–44	484 (47.6)	124 (44.3)	226 (46.4)	134 (53.6)
45–59	141 (13.9)	6 (2.1)	70 (14.4)	65 (26.0)
60+	58 (5.7)	0 (0)	33 (6.8)	25 (10.0)
Missing	1 (0.1)	0 (0)	1 (0.2)	0 (0)
ART reason				
CD4	549 (54.0)	0 (0)	376 (77.2)	173 (69.2)
PMTCT	280 (27.5)	280 (100.0)	0 (0)	0 (0)
WHO Stage	77 (7.6)	0 (0)	45 (9.2)	32 (12.8)
Test and treat	43 (4.2)	0 (0)	23 (4.7)	20 (8.0)
TB	39 (3.8)	0 (0)	17 (3.5)	22 (8.8)
Missing	29 (2.9)	0 (0)	26 (5.3)	3 (1.2)
ART start yr				
2014	211 (20.8)	58 (20.7)	101 (20.7)	52 (20.8)
2015	414 (40.7)	105 (37.5)	212 (43.5)	97 (38.8)
2016	350 (34.4)	107 (38.2)	157 (32.2)	86 (34.4)
2017	42 (4.1)	10 (3.6)	17 (3.5)	15 (6.0)
Time on ART				
≤3 mo	325 (32.0)	89 (31.8)	136 (27.9)	100 (40.0)
3–6 mo	190 (18.7)	70 (25.0)	88 (18.1)	32 (12.8)
6–12 mo	228 (22.4)	70 (25.0)	114 (23.4)	44 (17.6)
12–24 mo	219 (21.5)	39 (13.9)	120 (24.6)	60 (24.0)
>24 mo	55 (5.4)	12 (4.3)	29 (6.0)	14 (5.6)
Baseline CD4				
<100	206 (20.2)	14 (5.0)	106 (21.8)	86 (34.4)
100–199	185 (18.2)	38 (13.6)	87 (17.9)	60 (24.0)
200–349	261 (25.7)	71 (25.4)	129 (26.5)	61 (24.4)
350–499	193 (19.0)	74 (26.4)	95 (19.5)	24 (9.6)
500+	145 (14.3)	64 (22.9)	64 (13.1)	17 (6.8)
Missing	27 (2.6)	19 (6.8)	6 (1.2)	2 (0.8)
Baseline WHO stage				
I	722 (71.9)	261 (93.2)	329 (67.6)	132 (52.8)
II	143 (14.1)	17 (6.1)	73 (15.0)	53 (21.2)
III	129 (12.7)	2 (0.7)	70 (14.4)	57 (22.8)
IV	10 (1.0)	0 (0)	6 (1.2)	4 (1.6)
Missing	13 (1.3)	0 (0)	9 (1.8)	4 (1.6)
Refill schedule				
1 mo	672 (66.1)	188 (67.1)	322 (66.1)	162 (64.8)
2 mo	233 (22.9)	68 (24.3)	102 (20.9)	63 (25.2)
3 mo	79 (7.8)	20 (7.1)	44 (9.0)	15 (6.0)
>3 mo	33 (3.2)	4 (1.4)	19 (3.9)	10 (4.0)
Health facility				
Agincourt	272 (26.7)	74 (26.4)	141 (28.9)	57 (22.8)
Belfast	186 (18.3)	64 (22.9)	80 (16.4)	42 (16.8)
Cunningmore	58 (5.7)	16 (5.7)	32 (6.6)	10 (4.0)
Justicia	120 (11.8)	42 (15.0)	42 (8.6)	36 (14.4)
Kildare	117 (11.5)	25 (8.9)	62 (12.7)	30 (12.0)
Lillydale	166 (16.3)	32 (11.4)	81 (16.6)	53 (21.2)
Thulamahashe	25 (2.5)	9 (3.2)	12 (2.5)	4 (1.6)
Xanthia	73 (7.2)	18 (6.4)	32 (7.6)	18 (7.2)
Time since last appointment				
≤1 yr	526 (51.7)	130 (46.4)	255 (52.4)	141 (56.4)
1–2 yrs	369 (36.3)	117 (41.8)	176 (36.1)	76 (30.4)
>2 yrs	122 (12.0)	33 (11.8)	56 (11.5)	33 (13.2)
AHDSS outcome				
Still in HDSS	505 (49.7)	142 (50.7)	237 (48.7)	126 (50.4)
Deceased	74 (7.3)	6 (2.1)	42 (8.6)	26 (10.4)
Migrated	270 (26.5)	99 (35.4)	125 (25.7)	46 (18.4)
Not linked	168 (16.5)	33 (11.8)	83 (17.0)	52 (20.8)
Final outcome				
Deceased	120 (11.8)	10 (3.6)	60 (12.3)	50 (20.0)
Transferred out	315 (31.0)	82 (29.3)	176 (36.1)	57 (22.8)
Stopped ART	75 (7.4)	28 (10.0)	20 (4.1)	27 (10.8)
Migrated	49 (4.8)	21 (7.5)	22 (4.5)	6 (2.4)
Reengaged	225 (22.1)	54 (19.3)	110 (22.6)	61 (24.4)
Alive: ART unknown	111 (10.9)	45 (16.1)	45 (9.2)	21 (8.4)
LTFU	122 (12.0)	40 (14.3)	54 (11.1)	28 (11.2)

### Sources of Resolution

Of the 1017 patients LTFU, 895 (88.0%) were resolved, with 536 (59.9%) of these occurring through record review, 155 (17.3%) through demographic surveillance data (23 migrations, 21 deaths, 111 alive), 72 (8.0%) through subsequent visit data in the PIRL database, 53 (5.9%) through supplementary tracing, 57 (6.4%) identified as duplicates in the PIRL database (one person matching to multiple clinic records), and 22 (2.5%) through a search of patient records in clinics in close proximity to the patient's residence.

### Patient Outcomes

Of 1017 patients LTFU, 120 [11.8%, 95% confidence interval (CI): 9.9 to 13.9] had died, 315 (31.0%, CI: 28.1 to 33.9) had transferred to another facility, 75 (7.4%, CI: 5.8 to 9.1) had stopped ART, 49 (4.8%, CI: 3.6 to 6.3) had migrated, 225 (22.1%, CI: 19.6 to 24.8) re-engaged in care, 111 (10.9%, CI: 9.1 to 13.0) were alive with an unknown treatment status, and 122 (12.0%) remained LTFU. These outcomes differed (all *P* < 0.001) by sex, age, baseline CD4 count, time on ART, clinic visit schedule, health facility, time since a missed appointment, and ART initiation reason. Women who initiated treatment while pregnant or postpartum were less likely to have died [3.6% (CI: 1.7 to 6.5) compared with 14.9% (CI: 12.4 to 17.7)] and more likely to have migrated [7.5% (CI: 4.7 to 11.2) compared with 3.8% (CI: 2.5 to 5.4)], to be alive with their ART status unknown [16.1% (CI: 12.0 to 20.9) compared with 8.9% (CI: 7.0 to 11.2)] or stopped ART [10.0% (CI: 6.7 to 14.1) compared with 6.4% (CI: 4.7 to 8.4)] (Table 2).

**TABLE 2.** Patient Outcomes Disaggregated by Patient Demographic and Clinical Characteristics

	Outcome							Total
	Deceased	Transferred out	Stopped ART	Migrated	Reengaged	Alive: ART unknown	Still LTFU	All LTFU
	120	315	75	49	225	111	122	1017
	N (%)	N (%)	N (%)	N (%)	N (%)	N (%)	N (%)	
Sex (*P* < 0.001)								
Female	70 (9.1)	258 (33.6)	48 (6.3)	43 (5.6)	164 (21.4)	90 (11.7)	94 (12.2)	767 (75.4)
Male	50 (20.0)	57 (22.8)	27 (10.8)	6 (2.4)	61 (24.4)	21 (8.4)	28 (11.2)	250 (24.6)
Age (*P* < 0.001)								
18–29	17 (5.1)	117 (35.1)	24 (7.2)	25 (7.5)	61 (18.3)	46 (13.8)	43 (12.9)	333 (32.7)
30–44	55 (11.4)	147 (30.4)	37 (7.6)	21 (4.3)	116 (24.0)	50 (10.3)	58 (12.0)	484 (47.6)
45–59	27 (19.1)	38 (26.9)	11 (7.8)	2 (1.4)	35 (24.8)	13 (9.2)	15 (10.6)	141 (13.9)
60+	21 (36.2)	13 (22.4)	3 (5.2)	1 (1.7)	12 (20.7)	2 (3.4)	6 (10.3)	58 (5.7)
Missing	0 (0)	0 (0)	0 (0)	0 (0)	1 (100)	0 (0)	0 (0)	1 (0.1)
ART reason (*P* < 0.001)								
Non-PMTCT	110 (14.9)	233 (31.6)	47 (6.4)	28 (3.8)	171 (23.2)	66 (8.9)	82 (11.1)	737 (72.5)
PMTCT	10 (3.6)	82 (29.3)	28 (10.0)	21 (7.5)	54 (19.3)	45 (16.1)	40 (14.3)	280 (27.5)
ART start yr (*P* = 0.251)								
2014	28 (13.3)	58 (27.5)	14 (6.6)	18 (8.5)	50 (23.7)	19 (9.0)	24 (11.4)	211 (20.7)
2015	41 (9.9)	149 (36.0)	33 (8.0)	16 (3.9)	82 (19.8)	44 (10.6)	49 (11.8)	414 (40.7)
2016	46 (13.1)	100 (28.6)	24 (6.9)	14 (4.0)	82 (23.4)	41 (11.7)	43 (12.3)	350 (34.4)
2017	5 (11.9)	8 (19.0)	4 (9.5)	1 (2.4)	11 (26.2)	7 (16.7)	6 (14.3)	42 (4.1)
Time on ART (*P* < 0.001)								
≤3 mo	54 (16.6)	89 (27.3)	29 (8.9)	13 (4.0)	47 (14.5)	41 (12.6)	52 (16.0)	325 (32.0)
3–6 mo	18 (9.5)	62 (32.6)	13 (6.8)	8 (4.2)	31 (16.3)	30 (15.8)	28 (14.7)	190 (18.7)
6–12 mo	25 (11.0)	79 (34.6)	12 (5.3)	17 (7.5)	42 (18.4)	25 (11.0)	28 (12.3)	228 (22.4)
12–24 mo	16 (7.3)	76 (34.7)	17 (7.8)	9 (4.1)	75 (34.2)	13 (5.9)	13 (5.9)	219 (21.5)
>24 mo	7 (12.7)	9 (16.4)	4 (7.3)	2 (3.6)	30 (54.5)	2 (3.6)	1 (1.8)	55 (5.4)
Baseline CD4 (*P* < 0.001)								
<100	50 (24.3)	64 (31.1)	8 (3.9)	4 (1.9)	38 (18.4)	13 (6.3)	29 (14.1)	206 (20.2)
100–199	32 (17.3)	46 (24.9)	16 (8.6)	8 (4.3)	41 (22.2)	19 (10.3)	23 (12.4)	185 (18.2)
200–349	19 (7.3)	69 (26.4)	23 (8.8)	12 (4.6)	63 (24.1)	43 (16.5)	32 (12.3)	261 (25.7)
350–499	11 (5.7)	72 (37.3)	16 (8.3)	14 (7.3)	36 (18.6)	20 (10.4)	24 (12.4)	193 (19.0)
500+	8 (5.5)	53 (36.5)	11 (7.6)	10 (6.9)	41 (28.3)	12 (8.3)	10 (6.9)	145 (14.3)
Missing	0 (0)	11 (40.7)	1 (3.7)	1 (3.7)	6 (22.2)	4 (14.8)	4 (14.8)	27 (2.6)
Baseline WHO stage (*P* = 0.017)								
I	65 (9.0)	230 (31.8)	55 (7.6)	38 (5.3)	159 (22.0)	88 (12.2)	87 (12.0)	722 (71.0)
II	21 (14.7)	42 (29.4)	12 (8.4)	6 (4.2)	34 (23.8)	11 (7.7)	17 (11.9)	143 (14.1)
III	26 (20.1)	39 (30.2)	7 (5.4)	4 (3.1)	28 (21.7)	9 (7.0)	16 (12.4)	129 (12.7)
IV	5 (50.0)	1 (10.0)	1 (10.0)	0 (0)	2 (20.0)	0 (0)	1 (10.0)	10 (1.0)
Missing	3 (23.1)	3 (23.1)	0 (0)	1 (7.7)	2 (15.4)	3 (23.1)	1 (7.7)	13 (1.3)
Refill schedule (*P* < 0.001)								
1 mo	84 (12.5)	210 (31.2)	48 (7.1)	30 (4.5)	143 (21.3)	77 (11.4)	80 (11.9)	672 (66.1)
2 mo	24 (10.3)	71 (30.5)	21 (9.0)	14 (6.0)	43 (18.4)	24 (10.3)	36 (15.5)	233 (22.9)
3 mo	9 (11.4)	30 (38.0)	3 (3.8)	5 (6.3)	18 (22.8)	9 (11.4)	5 (6.3)	79 (7.8)
>3 mo	3 (9.1)	4 (12.1)	3 (9.1)	0 (0)	21 (63.6)	1 (3.0)	1 (3.0)	33 (3.2)
Health facility (*P* < 0.001)								
Agincourt	27 (9.9)	66 (24.3)	15 (5.5)	11 (4.0)	110 (37.1)	21 (7.7)	22 (8.1)	272 (26.7)
Belfast	16 (8.6)	52 (28.0)	13 (7.0)	12 (6.4)	32 (17.2)	29 (15.6)	32 (17.2)	186 (18.3)
Cunningmore	11 (19.0)	21 (36.2)	8 (13.8)	1 (1.7)	7 (12.1)	5 (8.6)	5 (8.6)	58 (5.7)
Justicia	20 (16.7)	30 (25.0)	13 (10.8)	7 (5.8)	14 (11.7)	11 (9.2)	25 (20.8)	120 (11.8)
Kildare	16 (13.7)	50 (42.7)	10 (8.5)	8 (6.8)	14 (12.0)	9 (7.7)	10 (8.5)	117 (11.5)
Lillydale	19 (11.4)	51 (30.7)	9 (5.4)	7 (4.2)	37 (22.3)	24 (14.5)	19 (11.4)	166 (16.3)
Thulamahashe	3 (12.0)	4 (16.0)	1 (4.0)	0 (0)	7 (28.0)	6 (24.0)	4 (16.0)	25 (2.4)
Xanthia	9 (12.2)	41 (55.4)	6 (8.1)	3 (4.0)	4 (5.4)	6 (8.1)	5 (6.8)	74 (7.3)
Time since last appointment (*P* < 0.001)								
≤1 yr	48 (9.1)	146 (27.8)	40 (7.6)	16 (3.0)	171 (32.5)	51 (9.7)	54 (10.3)	526 (51.7)
1–2 yrs	53 (14.4)	134 (36.3)	26 (7.0)	19 (5.1)	46 (12.5)	44 (11.9)	47 (12.7)	369 (36.3)
>2 yrs	19 (15.6)	35 (28.7)	9 (7.4)	14 (11.5)	8 (6.6)	16 (13.1)	21 (17.2)	122 (12.0)
AHDSS outcome (*P* < 0.001)								
Still in HDSS	17 (3.4)	177 (35.0)	52 (10.3)	7 (1.4)	141 (27.9)	111 (22.0)	0 (0)	505 (49.7)
Deceased	70 (94.6)	4 (5.4)	0 (0)	0 (0)	0 (0)	0 (0)	0 (0)	74 (7.3)
Migrated	22 (8.1)	86 (31.8)	19 (7.0)	34 (12.6)	58 (21.5)	0 (0)	51 (18.9)	270 (26.5)
Not linked	11 (6.5)	48 (28.6)	4 (2.4)	8 (4.8)	26 (15.5)	0 (0)	71 (42.3)	168 (16.5)

**FIGURE 1.**
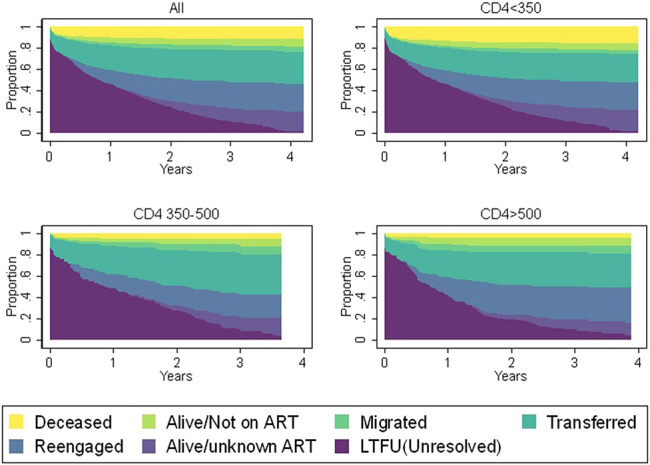
Status of patients by baseline CD4 and years since their last clinic visit.

**FIGURE 2.**
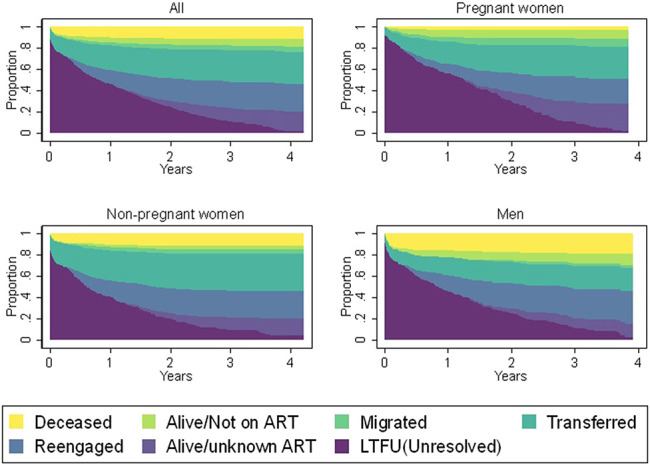
Status of patients by sex, pregnancy status at ART initiation, and years since their last clinic visit.

Most deaths occurred in the groups where baseline CD4 <200 cells/µL (Figure 1). Men were at highest risk of mortality, and pregnant women were at the lowest risk (Figure 2). Men and pregnant women also had higher risks of being alive and not in care compared with nonpregnant women (Figure 2). The mortality risk appeared to be similar for all CD4 categories for pregnant women unlike for nonpregnant women (see Supplementary Figure 4 and 5, Supplemental Digital Content, http://links.lww.com/QAI/B486). We also report on probable causes of death ascertained using VA data (see Supplementary information 2, Supplemental Digital Content, http://links.lww.com/QAI/B486).

### Factors Associated With Death

Of 120 deaths, 48 (37.2%) occurred before the patient's next visit date, 42 (32.6%) occurred after the patient's next scheduled visit date but before they would have met the criteria for LTFU, 30 (23.3%) occurred after the patient had met the criteria for LTFU, and 9 (7.0%) had a missing date of death.

In multivariable competing risk regression, being pregnant at ART initiation (aHR: 0.36, 95% CI: 0.15 to 0.87), and longer time on ART (12–24 months aHR: 0.44, 0.23 to 0.85) were associated with lower hazard of death following LTFU. Older age (≥60 years aHR: 8.86, 3.90–20.14) and lower CD4 at ART initiation (<100 cells/µL aHR: 3.77, 2.31–6.15; 100–199 cells/µL aHR: 2.35, 1.49–3.69) were associated with a higher hazard of death (Table 3).

**TABLE 3.** Factors Associated With Death

	HR (95% CI)	*P*	aHR (95% CI) n = 932	*P*
Sex				
Female	Reference	__		
Male	2.10 (1.57 to 2.81)	<0.001		
Age				
18–29	Reference	__	Reference	__
30–44	2.68 (1.30 to 5.51)	0.007	2.37 (0.98 to 5.75)	0.056
45–59	4.73 (3.13 to 7.15)	<0.001	2.96 (1.44 to 6.08)	0.003
60+	11.31 (5.32 to 24.06)	<0.001	8.86 (3.90 to 20.14)	<0.001
ART reason				
Non-PMTCT	Reference	__	Reference	__
PMTCT	0.17 (0.07 to 0.43)	<0.001	0.36 (0.15 to 0.87)	0.022
ART start yr				
2014	1.29 (0.82 to 2.04)	0.268		
2015	Reference	__		
2016	1.20 (0.67 to 2.14)	0.536		
2017	1.28 (0.83 to 1.97)	0.258		
Time on ART				
≤3 mo	Reference	__	Reference	__
3–6 mo	0.56 (0.31 to 0.99)	0.048	0.76 (0.46 to 1.25)	0.276
6–12 mo	0.74 (0.49 to 1.13)	0.167	0.82 (0.56 to 1.20)	0.307
12–24 mo	0.53 (0.31 to 0.91)	0.023	0.44 (0.23 to 0.85)	0.015
>24 mo	0.91 (0.41 to 2.05)	0.828	0.60 (0.23 to 1.56)	0.297
Baseline CD4				
<100	4.26 (3.11 to 5.82)	<0.001	3.77 (2.31 to 6.15)	<0.001
100–199	2.57 (1.60 to 4.12)	<0.001	2.35 (1.49 to 3.69)	<0.001
200–349	Reference	__	Reference	__
350–499	0.78 (0.39 to 1.55)	0.483	1.11 (0.53 to 2.36)	0.776
500+	0.82 (0.24 to 2.79)	0.756	1.13 (0.35 to 3.67)	0.840
Baseline WHO stage				
I	Reference	__	Reference	__
II	1.71 (0.98 to 3.00)	0.061	0.86 (0.40 to 1.86)	0.706
III	2.70 (1.77 to 4.14)	<0.001	1.36 (0.94 to 1.96)	0.102
IV	6.64 (3.08 to 14.32)	<0.001	3.14 (1.14 to 8.59)	0.026
Refill schedule				
1 mo	Reference	__		
2 mo	0.83 (0.37 to 1.86)	0.647		
3 mo	0.93 (0.49 to 1.75)	0.824		
>3 mo	0.74 (0.22 to 2.42)	0.615		
Health facility				
Agincourt	Reference	__	Reference	__
Belfast	1.03 (0.97 to 1.09)	0.345	0.80 (0.61 to 1.05)	0.108
Cunningmore	3.14 (2.98 to 3.31)	<0.001	3.39 (2.92 to 3.94)	<0.001
Justicia	2.10 (1.98 to 2.24)	<0.001	1.70 (1.55 to 1.86)	<0.001
Kildare	1.90 (1.84 to 1.95)	<0.001	1.08 (0.78 to 1.50)	0.639
Bhubezi	1.26 (1.19 to 1.34)	<0.001	0.96 (0.73 to 1.28)	0.810
Thulamahashe	0.93 (0.91 to 0.95)	<0.001	1.59 (1.15 to 2.22)	0.005
Xanthia	1.75 (0.70 to 1.80)	<0.001	1.98 (1.64 to 2.38)	<0.001
Time since last appointment				
≤1 yr	Reference	__	Reference	__
1–2 yrs	1.57 (1.03 to 2.39)	0.037	1.75 (1.10 to 2.78)	0.018
>2 yrs	1.65 (0.73 to 3.75)	0.228	0.81 (0.39 to 1.67)	0.564

All CD4 data was retrieved from clinic records (files and TIER.Net). All other clinical data was retrieved from the PIRL database (sex and age were crosschecked in clinic and HDSS records).

## DISCUSSION

We describe the treatment outcomes of HIV patients enrolled in care between April 2014 and August 2017 who had become LTFU in a rural South African setting as determined through a comprehensive record review and tracing study. Using multiple data sources and methods, we managed to ascertain the outcomes of 88% of the patients LTFU, a figure that is higher than most studies included in a recent systematic review of tracing studies in sub-Saharan Africa.^12^ We found that 31% of patients LTFU had transferred to another facility, 22% had re-engaged in care, and 12% of patients had died. These percentages varied by sex, reason for ART initiation, and baseline CD4 cell count. The differences for pregnant and postpartum women are particularly pertinent given that they represent the first iterations of treatment as prevention and could provide an indication for what to expect with the move to test and treat for all people living with HIV.

The proportion of patients reported as LTFU who had died in our study was substantially lower than the 42% and 39% reported in earlier systematic reviews of tracing studies from sub-Saharan Africa.^12,18^ Even if all the patients remaining LTFU after record review and tracing had died, mortality in our study would only rise to 24%. This lower percentage of deaths compared with the previous reviews is likely to be because of a healthier cohort of patients initiating treatment. We found that pregnant women were less likely to have died, an encouraging trend if it does translate to the general ART treatment population as less immunocompromised people begin to initiate ART. Mortality following LTFU may decrease further as universal test and treat policies result in growing proportions of asymptomatic patients initiating ART.

In competing risk survival analysis, being pregnant at ART initiation, higher baseline CD4, and longer time on ART were protective against death, whereas older age was found to be associated with a higher hazard of death following LTFU. Our findings suggest baseline CD4 cell count, WHO stage, and older age remain accurate measures for determining which patients are at highest risk for death,^42,44,45^ and these characteristics could be used to help prioritize tracing interventions. Whereas mortality risk appeared to wane with increasing CD4 at baseline for nonpregnant women and men, mortality appeared to be similar for all CD4 categories for women who initiated treatment for PMTCT. This may reflect the fact that their mortality risk was more influenced by other factors such as pregnancy related complications than by HIV.^46,47^ This could also be because of the fact that pregnant women were healthier in WHO staging compared with nonpregnant women and men, given the same CD4 at baseline, also reflected by the lower proportion of pregnant patients with a baseline CD4 <100 cells/µL. This discrepancy could also be related to temporary declines in CD4 count during pregnancy.^48^

Patients lost early on in treatment were at higher risk of death and this remained statistically significant even when controlling for baseline CD4, indicating that a longer duration on ART before attrition may reduce the risk of death. This protective effect appeared to be strongest for those who had been on ART 12–24 months before they became LTFU. This suggests that in settings with limited resources, tracing should be considered most urgent for newly ART-initiated patients who drop out of care. On the other hand, it may also indicate that some patients are still initiating treatment too late. In this study, 11% of nonpregnant patients had a CD4 cell count >500 cells/µL (compared with 23% of pregnant women), reflecting the fact that universal test and treat was not adopted in South Africa until September 2016.^49,50^ Men were disproportionately over-represented in the <200 cells/µL baseline CD4 category despite South African guidelines for ART initiation with CD4 <500 cells/µL having been in effect since January 2015.^51^ Men especially appear to be harder to reach and come into care later, similar to findings from other studies,^52–55^ and emphasizes the need to reach men earlier with ART.^56–58^

However, as the proportion of LTFU attributable to mortality dwindles, other outcomes are likely to become more prevalent. In our study, transfer to another facility accounted for 31% of patients who were reported as LTFU, which is higher than a previous systematic review.^12^ Other studies have suggested transfers become more common as programs expand and offer ART closer to patients' homes.^12,59,60^ Women were more likely to have transferred their care to another clinic. For pregnant women, this could reflect the higher mobility common during pregnancy and childbirth.^13,61,62^ Furthermore, given that most of these transfers were not reported to the sending facility similar to previous studies,^12,15^ these types of transfers could potentially lead to the spread of drug resistance in situations where ART experienced patients are offered regimens that have lost any therapeutic value due to drug resistance.^63^ Silent transfers may also lead to over-estimates of the number of people newly initiating ART and the number of people who have ever initiated ART. The current system of transferring patients could be improved by better referral systems, patient education, regular information exchange between clinics, and provider training.^64^

We found that 7.4% of patients had stopped treatment, with this being more common for women who initiated ART while pregnant, which adds to findings from previous studies that suggest that feeling healthy contributes to attrition for pregnant women.^65,66^ This figure is lower than the 28.6% of treatment interruptions reported in a recent systematic review.^12^ This may suggest that interventions to reduce interruptions, including routine tracing, are working well in this setting, further supported by the number of re-engagements in care that were observed in our study.

Our data showed that pregnant women and the general treatment cohort still differ significantly especially regarding immune system markers such as CD4. However, with the advent of test and treat, these groups may increasingly become similar in this regard and hence outcomes for pregnant women living with HIV could represent what treatment programs may expect to see in the future regarding patients that become LTFU especially those of a similar age. With ART programs in sub-Saharan Africa maturing, and with less immunologically compromised patients initiating ART, patients that become LTFU will be less likely to have died, whereas ART cessation or interruption and re-engagement in care are likely to become more common. Treatment programs will increasingly need to reallocate resources to deal with improving the clinic transfer process and invest in tracing and psychosocial support to get patients back in care or else risk having high community viral load which may increase the probability of onward transmission. We showed that 6% of patients who were late for a scheduled appointment returned before they officially became categorized as LTFU. These patients in theory would have received the routine tracing intervention offering further evidence of its utility, in line with a previous study that has highlighted how early active tracing of patients LTFU may improve patient outcomes and retention in care.^8^

Furthermore, given that most resolutions came through record review of tracing logbooks and clinic records, this study demonstrates that routine patient tracing still has utility for improving the completeness and accuracy of patient records. The availability of these data within the clinics suggests that routinely-collected data, especially those from the 2 organizations that assist in patient tracing needs to be better collated, integrated and recorded to ensure that patient outcomes are reflected in their clinic files and on TIER.Net. This study also demonstrates the utility of other data sources such as HDSS data. Given the push to integrate national ID numbers in patient profiles, clinics operating within similar health and demographic surveillance sites should consider liaising with these sites to improve the capture of deaths and migrations. Policy makers should also consider using South Africa's national death registry within clinics as this has been shown to be useful in other studies.^67,68^

This study had several limitations. First, the record review was cross-sectional; we only consulted clinic records at one point in time, whereas, some of these records may have subsequently been updated. Furthermore, we only consulted HBC and RtC logbooks that were afforded to us and it is possible that we might have missed some with information on patients we were trying to find. The observational nature of the study limited our ability to assess predictive factors and causality. We failed to ascertain the outcomes for 12% of our cohort and this may introduce some downward bias to our estimates. Finally, as we only resolved cause of death in 48.3% of patients found to have died, this data should be interpreted with caution. As we attempted to trace all adult patients LTFU, rather than a sample, these results are likely to be generalizable to other rural sub-Saharan settings. A strength of this study is the use of multiple data sources.

In conclusion, our study offers evidence for the growing utility for routine patient tracing. The different distribution of outcomes among Option B+ women suggests that different program mortality and attrition correction factors will be needed as universal test and treat becomes more established. Higher mortality among men emphasizes the importance of programmatic efforts to reach men earlier and treatment programs need to improve transfer procedures to make it more conducive for patients to move between clinics.

## Supplementary Material

SUPPLEMENTARY MATERIAL
