# Supplementary material for: Outcomes After Being Lost to Follow-up Differ for Pregnant and Postpartum Women When Compared With the General HIV Treatment Population in Rural South Africa
Source: J Acquir Immune Defic Syndr. 2020 Jun 10;85(2):127–37. doi: 10.1097/QAI.0000000000002413 (PMC7495979; doi:10.1097/QAI.0000000000002413)
Supplement: SUPPLEMENTARY MATERIAL [file qai-85-127-s001.docx]

**Supplementary materials:**

**Supplementary information 1:**

Of 188 patients who were categorised as data errors, 39 (20.7%) returned early (before their next scheduled visit), 58 (30.9%) returned on the exact date of their next scheduled visit, 30 (16.0%) were between 1 to 7 days late for their next scheduled visit, and 61 (32.4%) were more than 7 days late for their next scheduled visit and were a median of 43 days late (IQR: 27, 58). These 61 probably indicate the utility of routine tracing as tracing procedures would have in theory been performed for them. The 91 patients that were late for their scheduled visit, were late for a median of 27 days (IQR: 6, 55).

**Supplementary information 2: Causes of death.**

We managed to ascertain the probable cause of death for 58 (48.3%) of 120 deaths through verbal autopsy data from the HDSS. HIV/AIDS was indicated as the main cause of death for 10 (17.2%) deaths and acute respiratory infection for 11 (19.0%) deaths. Other causes included tuberculosis, malaria, cardiovascular disease, and neoplasms. These causes of death varied by ART initiation reason (Supplementary Table 1).

**Supplementary Table 1:** Probable causes of death as indicated by verbal autopsy data

|  | Pregnant women | Non-pregnant women | Men | Total |
| --- | --- | --- | --- | --- |
| Primary cause | **N (%)** | **N (%)** | **N (%)** | **N (%)** |
| Acute respiratory infection (including pneumonia) | 2 (20.0) | 6 (10.0) | 3 (6.0) | 11 (9.2) |
| HIV/AIDS related death | 0 (0) | 8 (13.3) | 2 (4.0) | 10 (8.3) |
| Pulmonary tuberculosis | 1 (10.0) | 0 (0) | 4 (8.0) | 5 (4.2) |
| Malaria | 0 (0) | 3 (5.0) | 0 (0) | 3 (2.5) |
| Oral neoplasms | 0 (0) | 0 (0) | 1 (2.0) | 1 (0.8) |
| Digestive neoplasms | 1 (10.0) | 0 (0) | 4 (8.0) | 5 (4.2) |
| Reproductive neoplasms | 0 (0) | 2 (3.3) | 0 (0) | 2 (1.7) |
| Severe malnutrition | 0 (0) | 1 (1.7) | 0 (0) | 1 (0.8) |
| Diabetes mellitus | 0 (0) | 0 (0) | 2 (4.0) | 2 (1.7) |
| Acute cardiac disease | 0 (0) | 1 (1.7) | 3 (6.0) | 4 (3.3) |
| Stroke | 0 (0) | 2 (3.3) | 0 (0) | 2 (1.7) |
| Other and unspecified cardiac disease | 0 (0) | 2 (3.3) | 1 (2.0) | 3 (2.5) |
| Asthma | 0 (0) | 2 (3.3) | 0 (0) | 2 (1.7) |
| Acute abdominal condition | 0 (0) | 1 (1.7) | 0 (0) | 1 (0.8) |
| Pregnancy induced hypertension | 0 (0) | 1 (1.7) | 0 (0) | 1 (0.8) |
| Obstetric haemorrhage | 1 (10.0) | 0 (0) | 0 (0) | 1 (0.8) |
| Pregnancy related sepsis | 0 (0) | 1 (1.7) | 0 (0) | 1 (0.8) |
| Anaemia of pregnancy | 1 (10.0) | 1 (1.7) | 0 (0) | 2 (1.7) |
| Accidental exposure to smoke fire and flames | 0 (0) | 1 (1.7) | 0 (0) | 1 (0.8) |
| No verbal autopsy data | 4 (40.0) | 28 (46.7) | 30 (60.0) | 62 (51.7) |
| Total | 10 | 60 | 50 | 120 |

**Supplementary Figure 1:** Record review and tracing flow chart


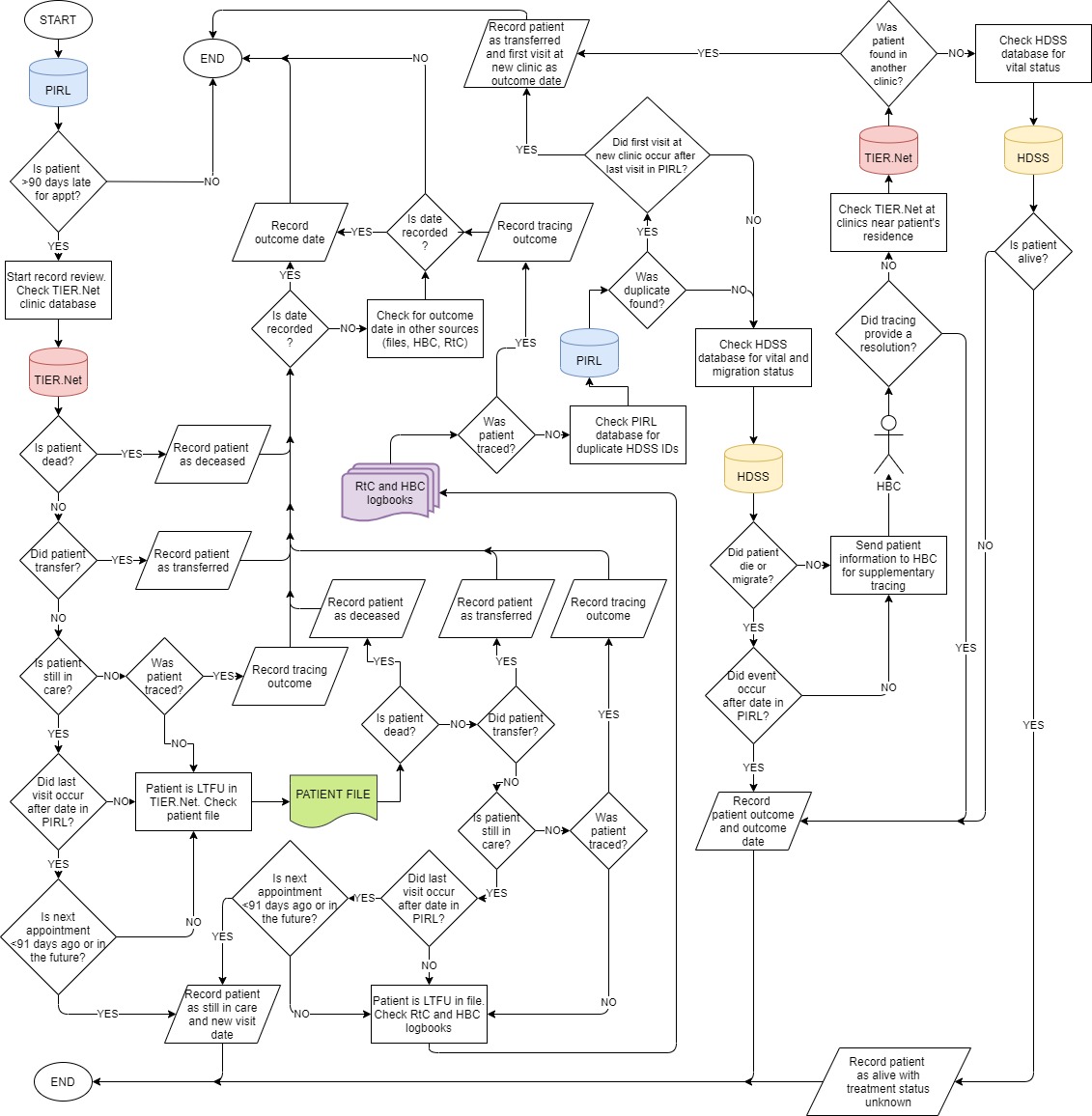


**Supplementary Figure 2:** Numbers of patients that were eligible at each stage and the number of patients excluded at each stage

**
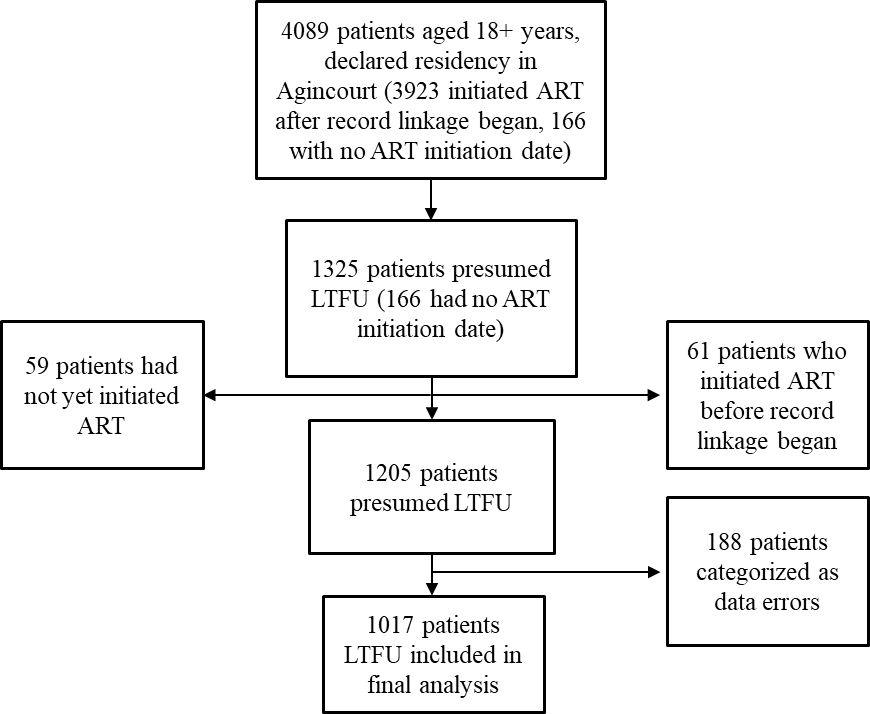
**

**Supplementary Figure 3:** A histogram of the distribution of days late for a missed appointment disaggregated by pregnancy status at ART initiation.

**Supplementary Figure 4:** Status of patients who were pregnant at ART initiation stratified by baseline CD4 count and years since their last clinic visit.

**Supplementary Figure 5:** Status of non-pregnant female patients stratified by baseline CD4 count and years since their last clinic visit.
